# Continuous blood pressure prediction system using Conv-LSTM network on hybrid latent features of photoplethysmogram (PPG) and electrocardiogram (ECG) signals

**DOI:** 10.1038/s41598-024-66514-y

**Published:** 2024-07-16

**Authors:** Bharindra Kamanditya, Yunendah Nur Fuadah, Nurul Qashri Mahardika T., Ki Moo Lim

**Affiliations:** 1 Department of IT Convergence Engineering, Kumoh National Institute of Technology, Gumi, 39177 Republic of Korea; 2 Department of Medical IT Convergence Engineering, Kumoh National Institute of Technology, Gumi, 39253 Republic of Korea; 3 Meta Heart Inc., Gumi, 39253 Republic of Korea; 4 Department of Electrical Engineering, Telkom University, Bandung, 40257 Indonesia

**Keywords:** Blood pressure, Photoplethysmography, Electrocardiography, Deep learning, LSTM, Hypertension, Health care, Diagnostic markers

## Abstract

Continuous blood pressure (BP) monitoring is essential for managing cardiovascular disease. However, existing devices often require expert handling, highlighting the need for alternative methods to simplify the process. Researchers have developed various methods using physiological signals to address this issue. Yet, many of these methods either fall short in accuracy according to the BHS, AAMI, and IEEE standards for BP measurement devices or suffer from low computational efficiency due to the complexity of their models. To solve this problem, we developed a BP prediction system that merges extracted features of PPG and ECG from two pulses of both signals using convolutional and LSTM layers, followed by incorporating the R-to-R interval durations as additional features for predicting systolic (SBP) and diastolic (DBP) blood pressure. Our findings indicate that the prediction accuracies for SBP and DBP were 5.306 ± 7.248 mmHg with a 0.877 correlation coefficient and 3.296 ± 4.764 mmHg with a 0.918 correlation coefficient, respectively. We found that our proposed model achieved a robust performance on the MIMIC III dataset with a minimum architectural design and high-level accuracy compared to existing methods. Thus, our method not only meets the passing category for BHS, AAMI, and IEEE guidelines but also stands out as the most rapidly accurate deep-learning-based BP measurement device currently available.

## Introduction

Monitoring BP is crucial in managing cardiovascular disease, particularly hypertension. This typically involves either invasive or non-invasive methods in clinical settings, each with unique challenges. Invasive arterial cannulation offers continuous monitoring but requires specialized expertise due to its complexity^1^. The more common non-invasive cuff-based method, while easier to use, is prone to inaccuracies from factors like patient movement and improper cuff sizing^2^. These limitations have driven the exploration of alternative monitoring methods.

Advancements in BP monitoring have focused on associating BP with other physiological parameters, thereby reducing the reliance on traditional measurement methods. Techniques that establish mathematical relationships between BP and pulse arrival time (PAT), pulse wave velocity (PWV), and pulse transit time (PTT) using the Moens–Korteweg and Bramwell–Hill physical models show promise. However, they still require recalibration for each individual, as noted in studies^3–6^. Bote et al.^3^ proposed a multivariate linear and inversely quadratic model for BP estimation, which incorporates heart rate variability to improve accuracy. Similarly, Gesche et al.^6^ developed a BP estimation method that calculates PWV by multiplying the body correction factor with height, then dividing by the PTT, and includes adjustments for individual variations.

Recent research has explored the use of machine learning algorithms for BP prediction utilizing ECG and PPG signals, eliminating the need for PAT or PTT calibrations, as noted in studies^7–10^. Previous studies have shown the effectiveness of machine learning models using only PPG signals. For instance, Xie et al^7^ predicted SBP and DBP from 10-s PPG signals using a Random Forest model. Similarly, Ali and Marco^8^ used 8-s PPG waveforms and demonstrated that deep learning architectures can achieve SBP and DBP predictions with lower error metrics. Although these methods do not involve PAT and PTT, they mark a significant advancement in non-invasive BP monitoring.

Further research has integrated both PPG and ECG signals into deep learning models, achieving greater accuracy. Annunziata et al.^9^ used various neural network architectures to predict SBP and DBP, as well as entire BP waveforms, finding that the combination of PPG and ECG signals resulted in lower errors than using PPG alone. Mahmud et al.^10^ employed a deep autoencoder architecture with a large number of parameters (more than 550,000) to extract features from ECG, PPG, and PPG derivatives for BP waveform prediction.

However, these studies have limitations. For example, Annunziata et al.^9^ used only 12 patients in their experiments, and the autoencoder used by Mahmud et al.^10^ required high computational resources, which might not be readily available for small devices. Similarly, innovative models by Baker et al.^11^ and Jeong and Lim^12^, which utilize temporal convolution and LSTM layers to process raw PPG and ECG waveforms, demonstrated varying success. These models also faced limitations in adhering to established BP measurement guidelines and computational efficiency. Baker et al.^11^ did not provide details on the parameter size of their deep model, while the model by Jeong and Lim^12^ was smaller in size, with only 38,370 parameters, but did not meet BP measurement guidelines due to the limited number of patients used in their experiments.

The use of PPG and ECG signals is appealing due to their non-invasive nature and user convenience. However, there is a lack of a BP prediction system with low computational time that meets the key standards of the the British Hypertension Society (BHS), the Association for the Advancement of Medical Instrumentation (AAMI), and the Institute of Electrical and Electronics Engineers Standards Association (IEEE-SA) while keeping its design simple. Our research aims to create a BP prediction system that fulfills these important criteria and has a streamlined, straightforward architecture.

Our approach merges features derived from two pulses of both ECG and PPG signals to predict SBP and DBP. By combining these signals, we aim to capture the PAT more accurately. We have optimized this method to reduce both the model size and computational time, thereby enhancing its efficiency and practicality. The results of our experiments demonstrate that our approach achieved high accuracy while maintaining robustness on the Medical Information Mart for Intensive Care (MIMIC) III dataset, offering a promising solution for BP monitoring challenges.

## Arterial blood pressure prediction method

**Figure 1:**
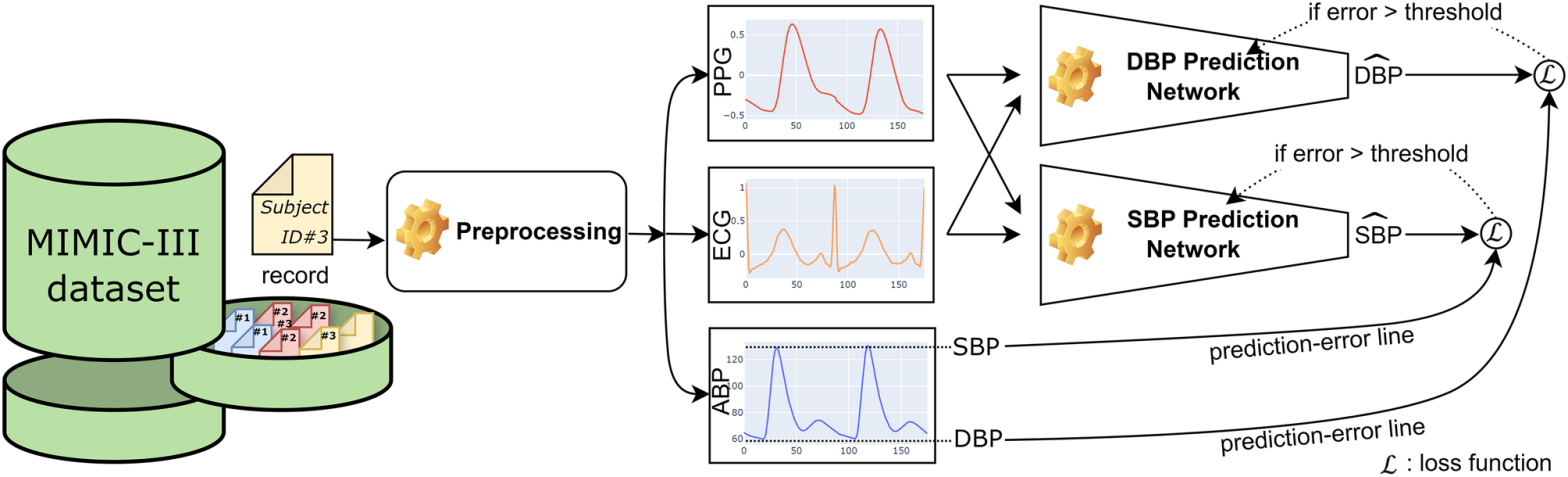
The flow diagram of our proposed method. The figure shows the process of predicting the systolic and diastolic blood pressure. Record files from the MIMIC-III dataset are passed to the preprocessing module. The output of the preprocessing module is three signals: PPG, ECG, and ABP. PPG and ECG signals are inputted to the DBP Prediction Network and SBP Prediction Network, whereas the ABP signal determines the target SBP and DBP values for training the Network.

The blood pressure prediction system proposed in this study comprises three main components, as illustrated in Fig. 1: Preprocessing, the SBP Prediction Network, and the DBP Prediction Network. The MIMIC III dataset undergoes preprocessing to obtain segmented PPG, ECG, and Arterial Blood Pressure (ABP) signals. The SBP and DBP values, extracted from the ABP signal, serve as target labels for the respective SBP and DBP Prediction Networks. In contrast, the PPG and ECG signals are employed as input data.

### Dataset and preprocessing

In this study, we used the MIMIC III dataset^13^ obtained from *Physionet* https://physionet.org/content/mimiciii to analyze the ABP, ECG, and PPG signals and determine the features for predicting SBP and DBP. The MIMIC III is an extensive dataset comprising various monitor trends of physiological measurements and waveforms, extending beyond ABP, PPG, and ECG, to include heart rate, respiratory rate, heart sounds, and numerous additional parameters. The dataset was collected from 38,597 patients at the Beth Israel Deaconess Medical Center in Boston, Massachusetts, encompassing their admissions exceeding 60 h in duration across five distinct Critical Care Units.

**Figure 2:**
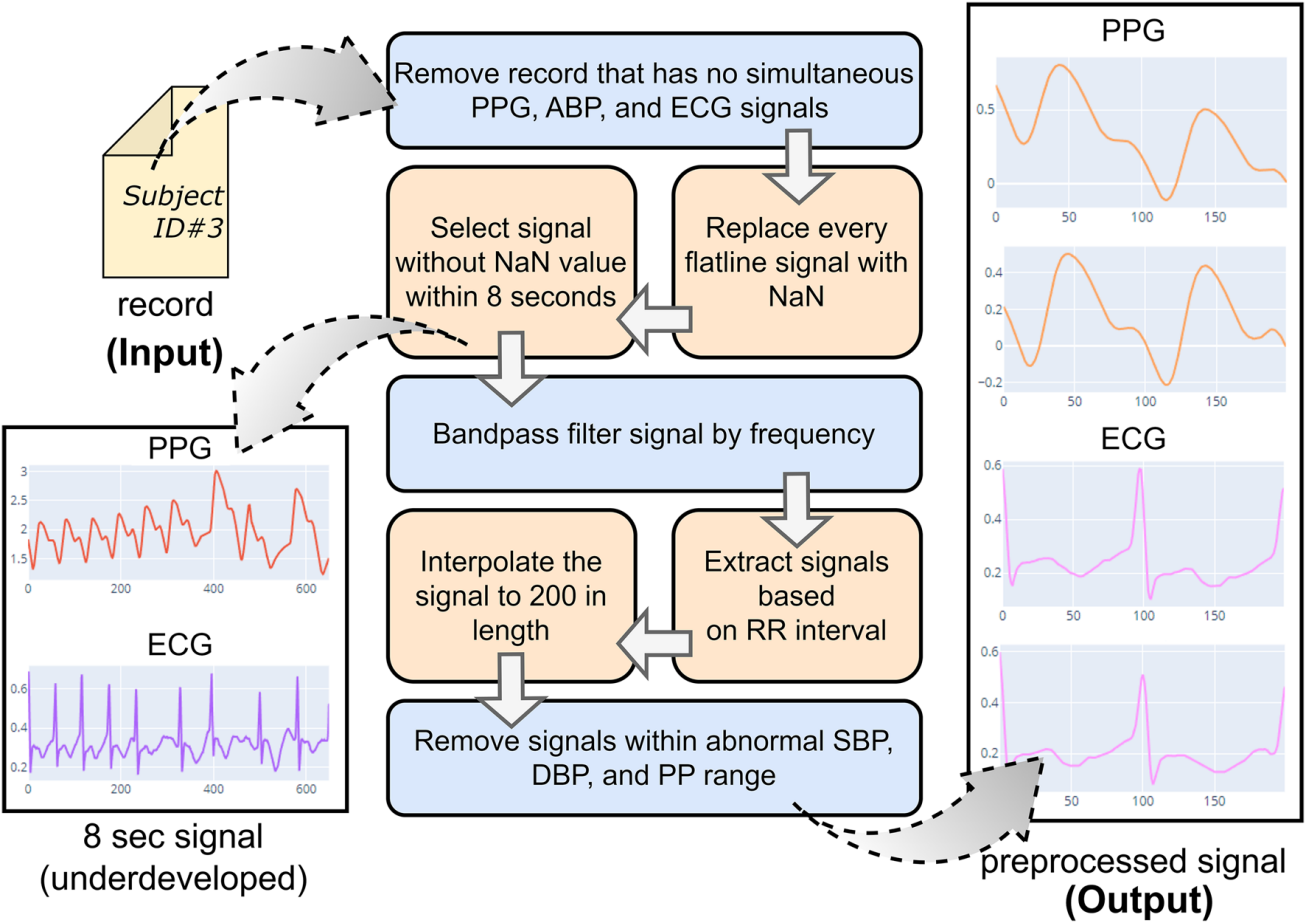
Preprocessing of the MIMIC III dataset used in our experiments. The figure shows the inside of the preprocessing module for extracting PPG and ECG signals. It consists of 7 consecutive blocks, with each block depicting a particular function.

Figure 2 presents the preprocessing stages for physiological signals. The first step involves selecting recordings that simultaneously include PPG, ABP, and ECG signals (specifically leads I, II, or III). Our proposed BP prediction system extracts the hybrid features from ECG and PPG, similar to the derivation of PAT and PTT features. The SBP and DBP values are determined from the peak and trough of the ABP signal, respectively.

In the second and third steps, we eliminate PPG and ECG signals that show constant or missing values over an 8-s interval. These signals, sampled at 125 Hz from the MIMIC III dataset, often have errors due to issues like sensor disconnections. The signals resulting from these steps are displayed in the lower left section of Fig. 2.

Next, we apply a bandpass filter (0.5 Hz to 20 Hz for PPG and 2 Hz to 20 Hz for ECG) to reduce motion artifacts and baseline wandering in the signals. We utilize the Pan-Tompkins algorithm^14^ to identify R peaks in the ECG signal, which enables the segmentation of the 8-s signals based on two R-to-R interval cycles. Subsequently, these segments are interpolated to a standard length of 200 points, ensuring consistent input for the prediction network.

Finally, we exclude PPG and ECG data if the corresponding ABP signal has SBP, DBP, or pulse pressure (PP) values outside normal ranges. PP is the difference between systolic and diastolic pressure. This exclusion applies to signals where SBP is above 200 mmHg or DBP is over 110 mmHg, indicating stage 3 hypertension, as well as signals with SBP below 90 mmHg and DBP below 50 mmHg. Additionally, we remove signals if the PP is above 70 mmHg, suggesting high blood pressure, or below 20 mmHg, which might indicate signs of heart failure. The processed PPG and ECG signals can be observed on the right side of Fig. 2.

### Design of the systolic and diastolic pressure prediction networks

We designed two identical deep-learning neural network architectures and trained them in a supervised manner to predict systolic and diastolic BP measurements. Both models used PPG and ECG signals, along with the duration of R-to-R intervals as input. Each respective model then calculates $\mathcal{L}$ as its error function, based on the differences between the predicted SBP $\left({\widehat{\mathbf{Y}}}_{\text{SBP}}\right)$ and DBP $\left({\widehat{\mathbf{Y}}}_{\text{DBP}}\right)$ values with the true SBP $\left({\mathbf{Y}}_{\text{SBP}}\right)$ and true DBP $\left({\mathbf{Y}}_{\text{DBP}}\right)$ values. The error function of the SBP and DBP prediction models can be written as:

1 $${\mathcal{L}}_{\left({\widehat{\mathbf{Y}}}_{\text{SBP}},{\mathbf{Y}}_{\text{SBP}}\right)}=\frac{1}{{\varvec{m}}}\sum_{{\varvec{t}}=1}^{{\varvec{m}}}{\left({\varvec{f}}\left({{\mathbf{X}}_{\text{PPG},\text{ECG},{\text{RR}}_{\text{intervals}}}}^{({\varvec{t}})}|\mathbf{W}\right)-{\mathbf{Y}}_{\text{SBP}}^{\left(t\right)}\right)}^{2}$$

2 $${\mathcal{L}}_{\left({\widehat{\mathbf{Y}}}_{\text{DBP}},{\mathbf{Y}}_{\text{DBP}}\right)}=\frac{1}{{\varvec{m}}}\sum_{{\varvec{t}}=1}^{{\varvec{m}}}{\left({\varvec{f}}\left({{\mathbf{X}}_{\text{PPG},\text{ECG},{\text{RR}}_{\text{intervals}}}}^{({\varvec{t}})}|\mathbf{W}\right)-{\mathbf{Y}}_{\text{DBP}}^{\left(t\right)}\right)}^{2}$$

with $f\left(\bullet \right)$ denotes the non-linearity function of the model, ${\mathbf{X}}_{\text{PPG},\text{ECG},{\text{RR}}_{\text{intervals}}}$ represents the inputs, i.e., preprocessed PPG and ECG signals and the R-to-R time duration(s), $\mathbf{W}$ denotes all the weight parameters of the model, $t$ is the $t$-th data sample and $m$ is the total number of samples.

**Figure 3:**
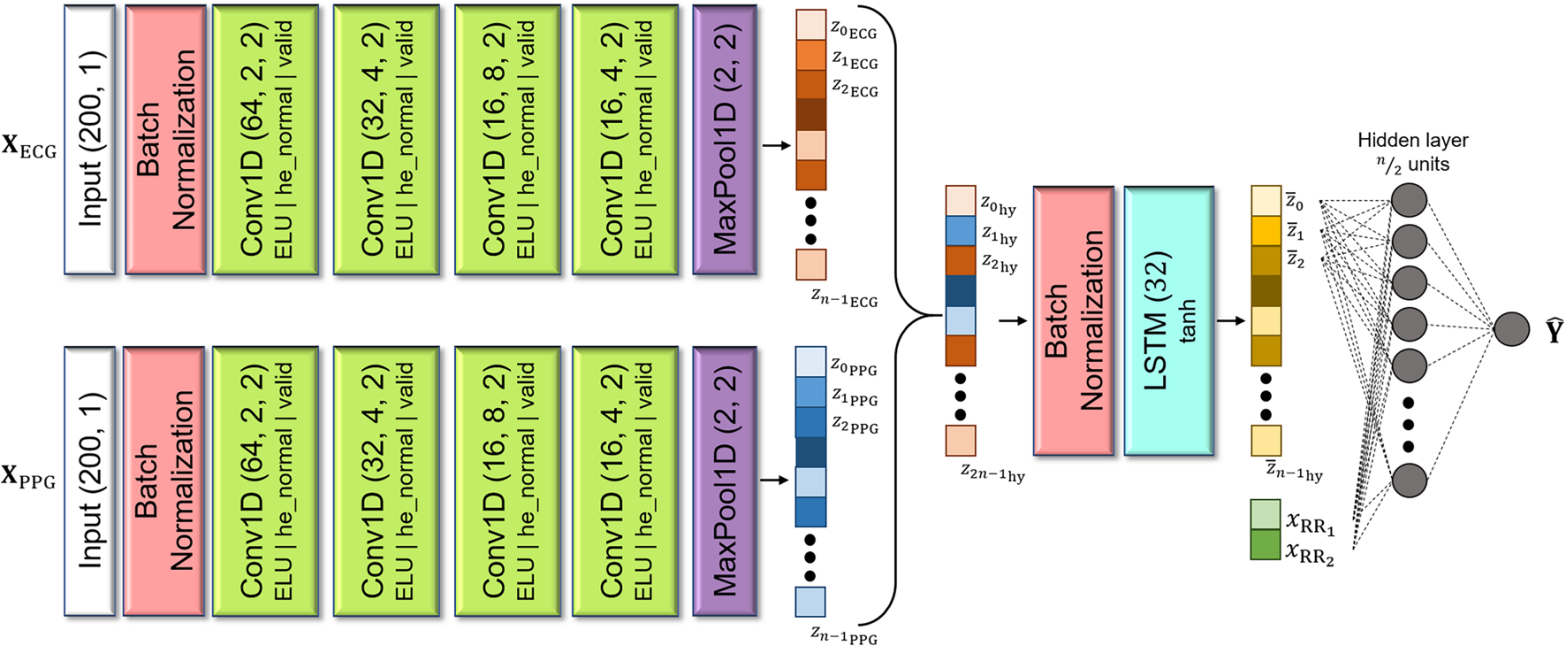
The SBP and DBP Prediction Networks. For predicting SBP, real SBP data was used as the target value. For predicting DBP, real DBP data was used. The network consists of Conv1D layers that process the ECG signal, Conv1D layers that process the PPG signal, a concatenation layer that mixes the features of PPG and ECG, an LSTM layer, and lastly, a fully connected layer to predict either SBP or DBP.

The process of predicting the desired output from the input signals is depicted in Fig. 3. A pair of fixed-length PPG and ECG signals are passed through four one-dimensional convolutional (Conv1D) layers. A single Conv1D layer slides several kernels across its input sequence to produce a 1D feature map per kernel. The number of kernels and the kernel size for each layer used in this network are as follows: (1st layer) 64 kernels with a size of 2, (2nd layer) 32 kernels with a size of 4, (3rd layer) 16 kernels with a size of 6, and (4th layer) 16 kernels with a size of 4. For every Conv1D layer, we used no padding and a stride of 2, which produced an output sequence always shorter than the input sequence, followed by a batch normalization layer and an activation function of exponential linear unit (ELU)^15^. Next, the feature maps undergo max pooling, producing latent feature representations of the input signals as vectors. We then concatenated the latent features of ECG, denoted as ${\mathbf{Z}}_{\text{ECG}}=\left({z}_{0}^{\text{ECG}},{z}_{1}^{\text{ECG}},\dots ,{z}_{n-1}^{\text{ECG}}\right)$ and PPG denoted as ${\mathbf{Z}}_{\text{PPG}}=\left({z}_{0}^{\text{PPG}},{z}_{1}^{\text{PPG}},\dots ,{z}_{n-1}^{\text{PPG}}\right)$ signals, as follows:

3 $${\mathbf{Z}}_{{{\text{hybrid}}}}^{\left( t \right)} = {\mathbf{Z}}_{{{\text{PPG}}}}^{\left( t \right)} +\!\!+ {\mathbf{Z}}_{{{\text{ECG}}}}^{\left( t \right)}$$

with $+\!\!+$ operator denotes the concatenation operator that outputs a hybrid latent features vector ${\mathbf{Z}}_{\text{hybrid}}=\left({z}_{0}^{\text{PPG}},{z}_{1}^{\text{PPG}},\dots ,{z}_{n-1}^{\text{PPG}},{z}_{0}^{\text{ECG}},{z}_{1}^{\text{ECG}},\dots ,{z}_{n-1}^{\text{ECG}}\right)$ with a length size of $2n$.

The long hybrid latent features vector is processed in an $n$-unit layer of long short-term memory (LSTM)^16^ cells following a batch normalization layer. A single LSTM cell functions as a network capable of detecting longer-term patterns in data by selectively retaining, accessing, and discarding information from the hybrid feature vector. The operation of the LSTM cell can be summarized, as follows:

4 $${\overline{\mathbf{Z}} }_{\left(t\right)}=\left({\mathbf{o}}_{\left(t\right)}|{\mathbf{Z}}_{\text{hybrid}}^{(t)},{\mathbf{h}}_{\left(t-1\right)},{\mathbf{W}}_{{\varvec{o}}}\right)\otimes \mathbf{t}\mathbf{a}\mathbf{n}\mathbf{h}\left({\mathbf{c}}_{\left(t\right)}|{\mathbf{Z}}_{\text{hybrid}}^{(t)},{\mathbf{c}}_{\left(t-1\right)},{\mathbf{h}}_{\left(t-1\right)},{\mathbf{f}}_{\left(t\right)},{\mathbf{g}}_{\left(t\right)},{\mathbf{r}}_{\left(t\right)}\right)$$

With ${\mathbf{Z}}_{\text{hybrid}}^{(t)}$ as the LSTM units’ current input, namely hybrid feature vector at time $t$, ${\overline{\mathbf{Z}} }_{\left(t\right)}$ is defined as the LSTM output vector with a length of $n$. The symbols ${\mathbf{c}}_{\left(t-1\right)}$ and ${\mathbf{h}}_{\left(t-1\right)}$ represent the long-term and the short-term states from the previous time step, respectively. Hyperbolic tangent $\text{tanh}\left(\bullet \right)$ was selected as the activation function of the LSTM cell to mitigate the unstable gradients problem. The states at the current time step, ${\mathbf{c}}_{\left(t\right)}$ and ${\mathbf{h}}_{\left(t\right)}$, where ${\mathbf{h}}_{\left(t\right)}={\overline{\mathbf{Z}} }_{\left(t\right)}$, utilize ${\mathbf{g}}_{\left(t\right)}$ as the primary source of information and are regulated by three gates: the *forget gate* ${\mathbf{f}}_{\left(t\right)}$, the *read gate* ${\mathbf{r}}_{\left(t\right)}$, and the *output gate* ${\mathbf{o}}_{\left(t\right)}$. ${\mathbf{g}}_{\left(t\right)},{\mathbf{f}}_{\left(t\right)},{\mathbf{o}}_{\left(t\right)},{\mathbf{r}}_{\left(t\right)}$ are generated by four separate fully connected networks from ${\mathbf{Z}}_{\text{hybrid}}^{(t)}$ and ${\mathbf{h}}_{\left(t-1\right)}$, with ${\mathbf{W}}_{o}$ denoting the weight matrices of the network that produces ${\mathbf{o}}_{\left(t\right)}$.

Assuming the hyperparameter ‘$n$’ in the SBP and DBP Prediction Networks is set to $n=32$, the hybrid feature vector of the ECG and PPG signals is then used as the input for the hidden layer to predict BP. The vector ${\overline{\mathbf{Z}} }_{\left(t\right)}$ and additional R-to-R duration intervals ${\mathbf{X}}_{{\text{RR}}_{\text{intervals}}}=\left({x}_{{\text{RR}}_{1}},{x}_{{\text{RR}}_{2}}\right)$ were combined and subjected to a *dropout* rate of 0.2 before being input into a hidden layer comprising 16 neurons, followed by a single output neuron to predict ${\widehat{\mathbf{Y}}}_{\left(t\right)}$. Finally, ${\widehat{\mathbf{Y}}}_{\left(t\right)}$ can be written as:

5 $${\hat{\mathbf{Y}}}_{{\left( {\varvec{t}} \right)}} = {\mathbf{softplus}}\left( {{\mathbf{ELU}}\left( {\left( {{\overline{\mathbf{Z}}}_{{\left( {\varvec{t}} \right)}} +\!\!+ {\varvec{x}}_{{{\mathbf{RR}}_{1} }}^{{\left( {\varvec{t}} \right)}} +\!\!+ {\varvec{x}}_{{{\mathbf{RR}}_{2} }}^{{\left( {\varvec{t}} \right)}} } \right){\mathbf{W}}_{{\mathbf{h}}} + {\mathbf{b}}_{{\mathbf{h}}} } \right){\mathbf{W}}_{{{\mathbf{out}}}} + {\mathbf{b}}_{{{\mathbf{out}}}} } \right)$$

Here, ${\mathbf{W}}_{\text{h}}$ and ${\mathbf{W}}_{\text{out}}$ denote the weight matrices of the hidden and output layers, respectively. Similarly, the row vectors ${\mathbf{b}}_{\text{h}}$ and ${\mathbf{b}}_{\text{out}}$ represent the bias terms for each layer. Functions $\text{ELU}\left(\bullet \right)$ and $\text{softplus}\left(\bullet \right)$ represent the activation function of the hidden and output layers, respectively.

### Experimental setups and standards of evaluation

**Figure 4:**
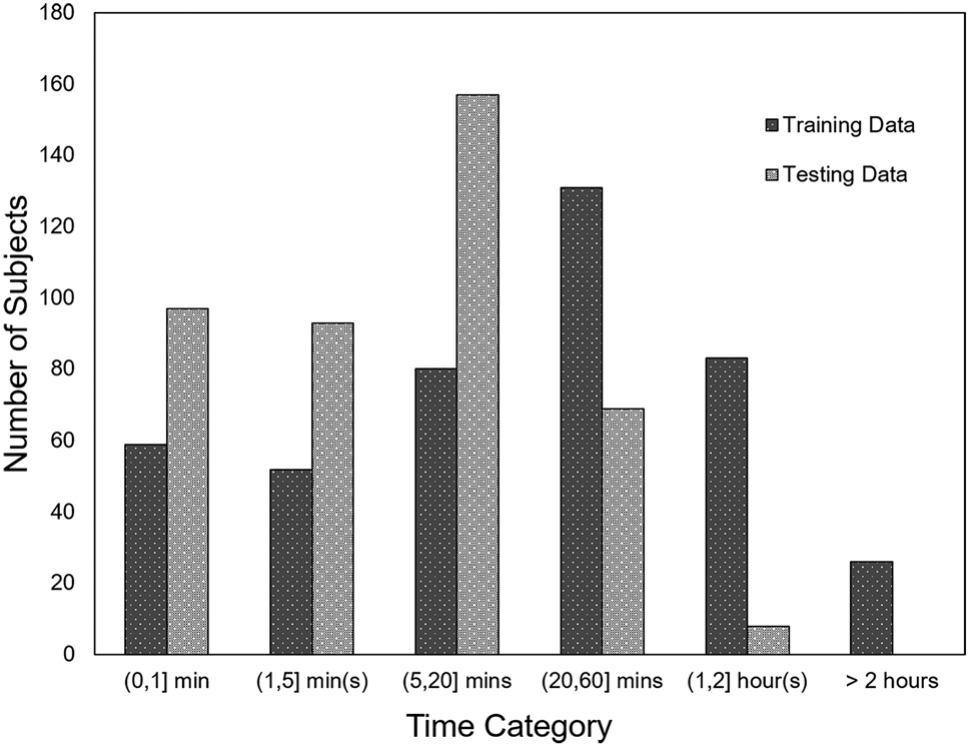
Distribution of subjects based on the total signal duration for the Training and Testing Datasets. A clustered column plot with a y-axis showing total the number of subjects belonging to a time duration category and an x-axis showing the duration length categories.

**Figure 5:**
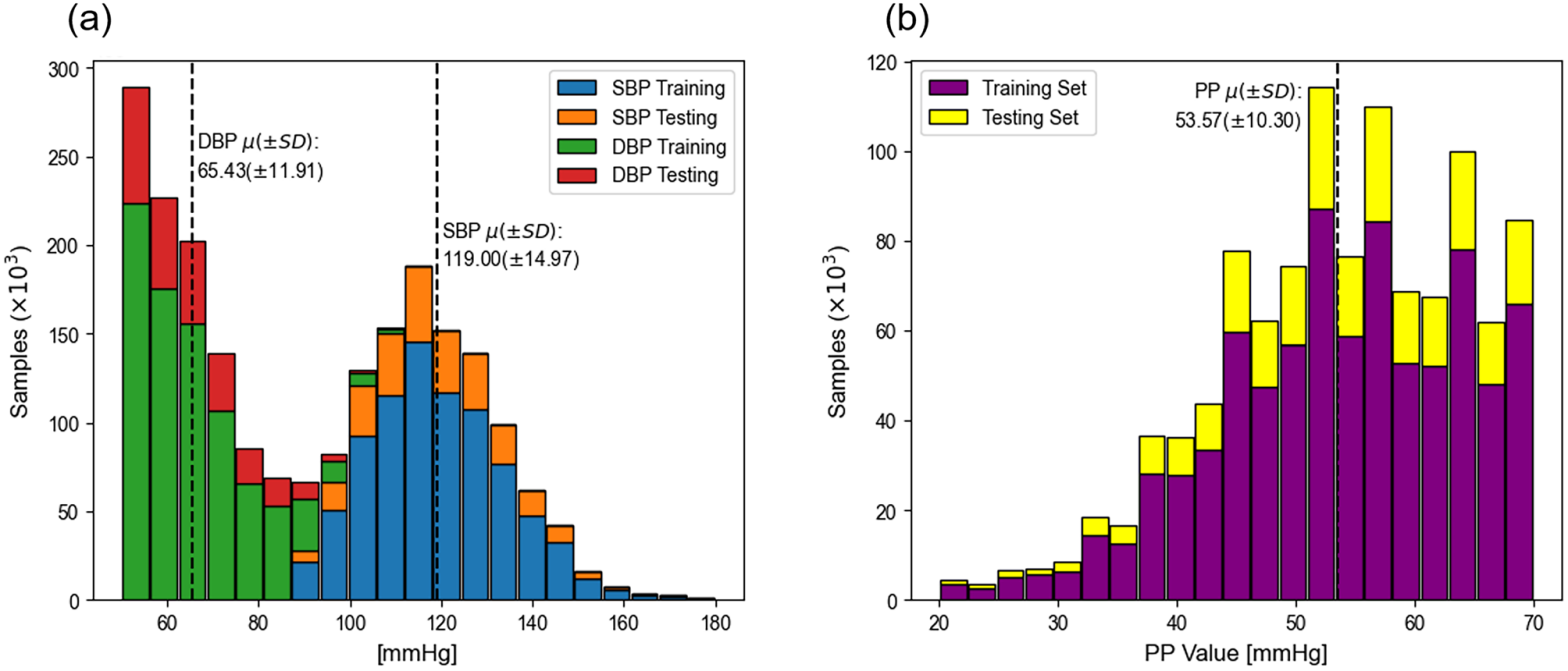
Distribution of blood pressure values in the Training and Testing datasets. The SBP and DBP distributions of the Training and Testing datasets are shown in panel (**a**), and the distributions of calculated PP for the Training and Testing datasets are shown in panel (**b**). The distributions are shown in a vertically stacking bar plot for both panels (**a**) and (**b**) with the y-axis showing the number of samples and the x-axis showing the blood pressure value in mmHg.

We randomly selected 431 distinct subjects in the MIMIC III database and collected a total of 1,079,529 samples after the Preprocessing. We then divided the samples from each subject into two datasets: a training dataset containing 831,335 (77%) of the total samples, and a testing dataset comprising the remaining 248,194 (23%) samples. Figure 4 displays a histogram showing the distribution of subjects based on the total signal duration length in our experiments. Figure 5 depicts the distributions of SBP and DBP in panel (a), and PP distribution in panel (b) of the two datasets. Figure 5 panel (a) shows that for SBP predictions, the minimum and the maximum values of the distribution were 90 mmHg and 180 mmHg, respectively, with a mean of 119.00 ± 14.97 mmHg. For DBP predictions, the minimum and the maximum values of the distribution were 50 mmHg and 110 mmHg, respectively, with a mean of 65.43 ± 11.91 mmHg. Figure 5 panel (b) shows that the mean of the calculated PP distribution was 53.57 ± 11.91 mmHg. These wide range of BP values were used on both training dataset and testing dataset to prove the robustness of our proposed model architectures.

The experimental setup was designed to investigate the performance of LSTM cells and R-to-R duration intervals as features in predicting BP. The experiment involved training four different model architectures on the training dataset: layered-Conv1D with and without R-to-R duration features, and Conv1D-LSTM with and without R-to-R duration features. This was done twice, first using SBP as the target data, and then using DBP. The prediction performance of each model was then measured on the testing dataset for both types of target data. The training dataset was divided into 10 sub-datasets for tenfold cross-validation. Each model was trained using these sub-datasets, and the model demonstrating the best performance on the testing set was selected as the experimental result.

The proposed model was evaluated using five metric formulas: Pearson’s correlation coeeficient ($r$), the mean difference (MD), the mean absolute error (MAE), the standard deviation (SD) of the error, and the groups of cumulative percentages (CP) for calculated errors less than or equal to 5, 10, and 15 mmHg. The $r$, MD, MAE, SD, and CP can be written as:

6 $${\varvec{r}}=\frac{1\sum_{{\varvec{t}}=1}^{{\varvec{m}}}\left({\mathbf{Y}}_{\left({\varvec{t}}\right)}-\overline{\mathbf{Y} }\right)\left({\widehat{\mathbf{Y}}}_{\left({\varvec{t}}\right)}-\overline{\widehat{\mathbf{Y}}}\right)}{\sqrt{\sum_{{\varvec{t}}=1}^{{\varvec{m}}}{\left({\mathbf{Y}}_{\left({\varvec{t}}\right)}-\overline{\mathbf{Y} }\right)}^{2}}\sqrt{\sum_{{\varvec{t}}=1}^{{\varvec{m}}}{\left({\widehat{\mathbf{Y}}}_{\left({\varvec{t}}\right)}-\overline{\widehat{\mathbf{Y}}}\right)}^{2}}}$$

7 $${\varvec{M}}{\varvec{D}}=\frac{1}{{\varvec{m}}}\sum_{{\varvec{t}}=1}^{{\varvec{m}}}\left({\widehat{\mathbf{Y}}}_{\left({\varvec{t}}\right)}-{\mathbf{Y}}_{\left({\varvec{t}}\right)}\right)$$

8 $${\varvec{M}}{\varvec{A}}{\varvec{E}}=\frac{1}{{\varvec{m}}}\sum_{{\varvec{t}}=1}^{{\varvec{m}}}\left|{\widehat{\mathbf{Y}}}_{\left({\varvec{t}}\right)}-{\mathbf{Y}}_{\left({\varvec{t}}\right)}\right|$$

9 $${\varvec{S}}{\varvec{D}}=\sqrt{\frac{1}{{\varvec{m}}}\sum_{{\varvec{t}}=1}^{{\varvec{m}}}{\left(\left({\widehat{\mathbf{Y}}}_{\left({\varvec{t}}\right)}-{\mathbf{Y}}_{\left({\varvec{t}}\right)}\right)-{\varvec{M}}{\varvec{D}}\right)}^{2}}$$

10 $${{\varvec{C}}{\varvec{P}}}_{\mathbf{m}\mathbf{m}\mathbf{H}\mathbf{g}\le \left[5,10,15\right]}=\frac{100\boldsymbol{\%}}{{\varvec{m}}}\times \sum_{{\varvec{t}}=1}^{{\varvec{m}}}{\left|{\widehat{\mathbf{Y}}}_{\left({\varvec{t}}\right)}-{\mathbf{Y}}_{\left({\varvec{t}}\right)}\right|}_{\le \left[5,10,15\right]}$$

The evaluation metrics are used as grading criteria to meet the standards established by the BHS^17^, the AAMI^18^, and the IEEE-SA^19^ as illustrated in Table 1. The BHS grades the performance of the model into categories based on the obtained CP metrics: A grade if CPmmHg ≤ 5 ≥ 60%, CPmmHg ≤ 10 ≥ 85%, and CPmmHg ≤ 15 ≥ 95%, B grade if CPmmHg ≤ 5 ≥ 50%, CPmmHg ≤ 10 ≥ 75%, and CPmmHg ≤ 15 ≥ 90%, C grade if CPmmHg ≤ 5 ≥ 40%, CPmmHg ≤ 10 ≥ 65%, and CPmmHg ≤ 15 ≥ 85%, and a D grade if performed worse than C. The AAMI recommends that the MD and the SD shall be ≤ 5 mmHg and ≤ 8 mmHg, respectively. The IEEE standards grade the performance based on the obtained MAE metric value: A grade if MAE score ≤ 5 mmHg, B grade if MAE = 5–6 mmHg, C grade if MAE = 6–7 mmHg, D grade if MAE ≥ 7 mmHg.

**Table 1:** Grading criteria defined by the BHS, AAMI, and IEEE standards.

BHS				AAMI			IEEE	
Grade	CP Δx ≤ 5 mmHg	CP Δx ≤ 5 mmHg	CP Δx ≤ 5 mmHg%	Grade	MD (mmHg)	SD (mmHg)	Grade	MAE (mmHg)
A	60%	85%	95%	Pass	≤ 5	≤ 8	A	≤ 5
B	50%	75%	90%				B	5–6
C	40%	65%	85%	Fail	MD and/or SD higher than the Pass category		C	6–7
D	Lower than C						D	> 7

Metrics include mean difference (MD), mean absolute error (MAE), standard deviation (SD), and cumulative percentage (CP).

## Experimental results

### Performance analysis

Tables 2 and 3 show the results of using Conv1D-LSTM and layered Conv1D models for predicting SBP and DBP, respectively. The Conv1D-LSTM model demonstrates lower errors and higher CP across all categories, including MAE, SD, and CPmmHg ≤ 5, CPmmHg ≤ 10, and CPmmHg ≤ 15, compared to the layered Conv1D model. Table 2 reveals that the Conv1D-LSTM method, without incorporating R-to-R duration intervals in the hybrid latent features vector, achieved the lowest MAE and SD for SBP prediction–5.306 mmHg and 7.248 mmHg, respectively. This model attains 59.823%, 85.919%, and 94.925% in CPmmHg ≤ 5, CPmmHg ≤ 10, and CPmmHg ≤ 15, respectively. On the other hand, Table 3 indicates that for DBP prediction, the lowest MAE and SD scores–3.296 mmHg and 4.764 mmHg–are obtained by the Conv1D-LSTM method by incorporating R-to-R duration intervals in the hybrid latent features vector, with 80.041%, 95.418%, and 98.613% in CPmmHg ≤ 5, CPmmHg ≤ 5, and CPmmHg ≤ 15. According to our evaluation criteria, the top model configurations used in our experiments achieved a B grade for SBP predictions and an A grade for DBP predictions in the BHS guidelines, passed the AAMI recommendation, and received a B grade and an A grade in the IEEE-SA Standards for the SBP and DBP predictions, respectively.

**Table 2:** Performances of SBP prediction networks used in our experiments.

Model architecture	Blood pressure error (mmHg)			Cumulative percentage (%)			Standards of evaluation		
	MD	MAE	SD	Δx ≤ 5 mmHg	Δx ≤ 10 mmHg	Δx ≤ 15 mmHg	BHS grade	AAMI grade	IEEE grade
Conv1D	0.774	6.061	8.076	53.143	81.489	92.924	B	Pass	C
Conv1D with R-to-R features	0.074	5.962	7.998	53.936	82.218	93.304	B	Pass	B
Conv1D-LSTM	− 0.503	5.306	7.248	59.823	85.919	94.925	B	Pass	B
Conv1D-LSTM with R-to-R features	0.031	5.317	7.263	59.634	86.001	94.970	B	Pass	B

Various model architectures are measured using mean absolute error (MAE), standard deviation (SD), and cumulative percentages (CP) metrics and graded based on standards of evaluation by BHS, AAMI, and IEEE.

**Table 3:** Performances of DBP prediction networks used in our experiments.

Model architecture	Blood pressure error (mmHg)			Cumulative percentage (%)			Standards of evaluation		
	MD	MAE	SD	Δx ≤ 5 mmHg	Δx ≤ 10 mmHg	Δx ≤ 15 mmHg	BHS grade	AAMI grade	IEEE grade
Conv1D	− 0.132	3.812	5.397	74.117	93.649	98.073	A	Pass	A
Conv1D with R-to-R features	0.049	3.767	5.335	74.942	93.778	98.128	A	Pass	A
Conv1D-LSTM	− 0.186	3.328	4.809	79.632	95.213	98.576	A	Pass	A
Conv1D-LSTM with R-to-R features	− 0.162	3.296	4.764	80.041	95.418	98.613	A	Pass	A

The results of experiments in this table are evaluated the same way as in Table 2.

Including R-to-R duration intervals in the hybrid feature vector led to a reduced MAE for DBP prediction but a slight increase for SBP prediction in the Conv1D-LSTM model. Specifically, for SBP predictions, the MAE decreased by 0.099 mmHg in the layered Conv1D model and increased by 0.011 mmHg in the Conv-LSTM model. Whereas for DBP predictions, the MAE decreased by 0.032 mmHg in the layered Conv1D model and the Conv1D-LSTM model.

**Figure 6:**
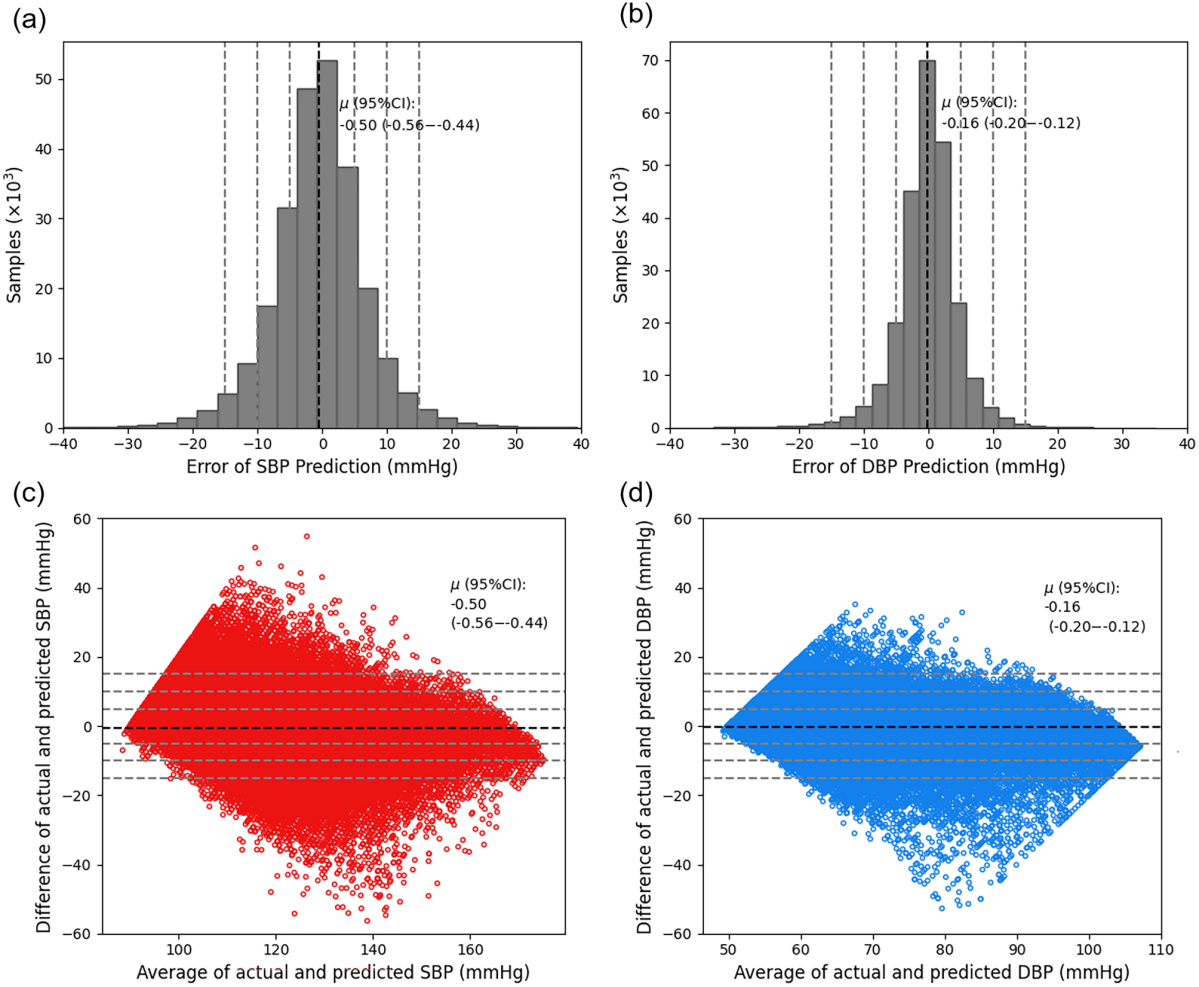
Error distributions and the Bland–Altman plots of the predicted SBP and DBP by the proposed model: (**a**) Error histogram for SBP, (**b**) Error histogram for DBP, (**c**) Bland–Altman plot for SBP, and (**d**) Bland–Altman plot for DBP. Figure panel (**a**) and (**b**) show an unimodal distribution of prediction error with the mean shown in bold-dashed lines. Panel (**c**) and (**d**) show scattered points of prediction data, shown in red color for SBP and blue color for DBP in the Bland–Altman plot, which shows the difference between the error of the predicted measurements against the averages of the error.

Figure 6 depicts the accuracy criteria for our experimental results, showing error distributions in histograms and Bland–Altman plots for SBP and DBP predictions using Conv1D-LSTM models, both without and with R-to-R duration intervals in the hybrid latent feature vector. In Fig. 6 panel (a), a histogram of SBP prediction errors is shown with a mean (µ) of − 0.50 and a 95% confidence interval (95%CI) ranging from − 0.56 to − 0.44. Figure 6 panel (b) displays a histogram of DBP prediction errors with a mean (µ) of − 0.16 and a 95%CI from − 0.20 to − 0.12. The reference grey dashed lines show the ± 5, ± 10, and ± 15 mmHg differences. Figures 6 panel (c) and panel (d) illustrate Bland–Altman plots of the differences between actual and predicted values against their mean values for SBP and DBP predictions, respectively. Figure 6 panel (c), corresponding to the best results in Table 2, shows that 59.82% of data points fall within ± 5 mmHg, 85.92% are within ± 10 mmHg, and 94.93% within ± 15 mmHg error lines. Figure 6 panel (d), aligning with the best results in Table 3, indicates that 80.04% of data points fall within ± 5 mmHg, 95.42% within ± 10 mmHg, and 98.61% within ± 15 mmHg error lines.

**Figure 7:**
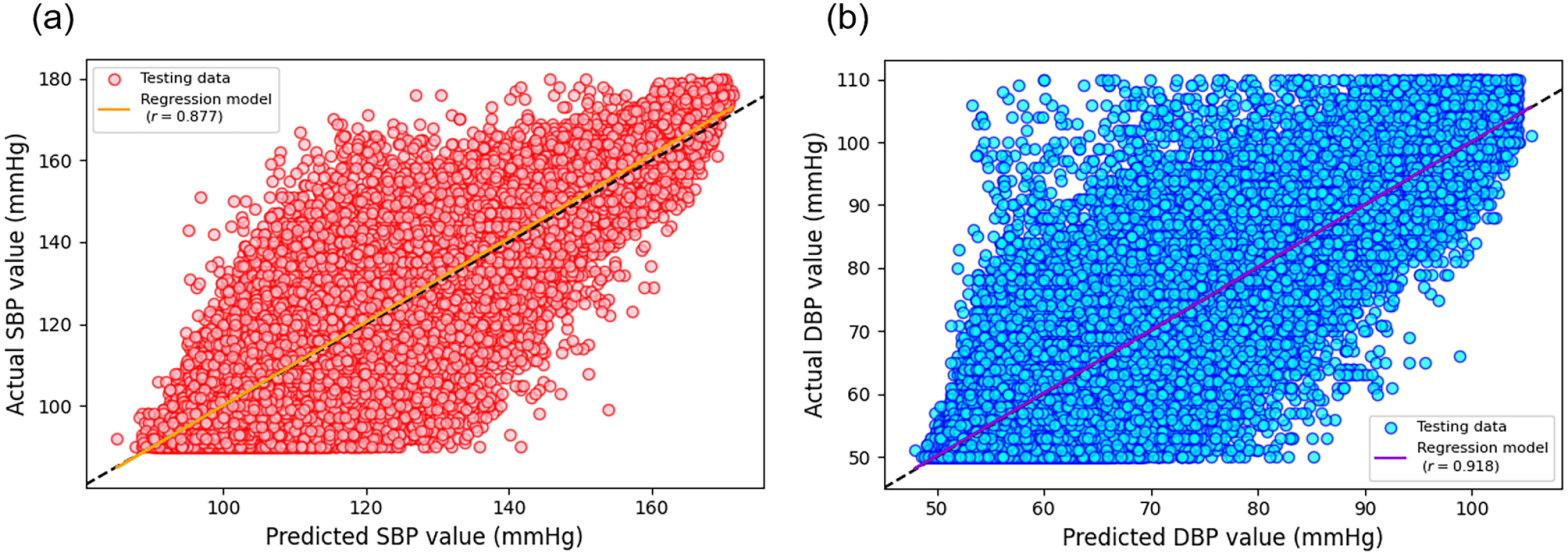
Distribution of subjects based on the total signal duration for the Training and Testing Datasets. Panel (**a**) shows a scatterplot of actual versus predicted SBP values from Conv1D-LSTM without R-to-R intervals. Panel (**b**) shows a scatterplot of actual versus predicted DBP values from Conv1D-LSTM with R-to-R intervals. Each plot has a regression line, orange for SBP and purple for DBP. This line overlays a dashed black line indicating perfect predictions.

Figure 7 panel (a) displays a scatterplot of the actual versus predicted SBP values using Conv1D-LSTM with R-to-R duration intervals, while Fig. 7 panel (b) presents a scatterplot for the actual versus predicted DBP values from Conv1D-LSTM without R-to-R duration intervals. Each scatterplot includes a regression line–orange for SBP and purple for DBP–that overlays a dashed black line representing a perfect prediction. These plots demonstrate a strong positive Pearson’s correlation between actual and predicted values, with $r$ = 0.877 for SBP predictions and $r$ = 0.918 for DBP predictions.

### Comparisons with previous works

The best Conv1D-LSTM models for predicting SBP and DBP, both without and with R-to-R duration intervals, were selected to compare our method’s performance with existing methods. Table 4 presents the performance of our method alongside other deep-learning-based blood pressure prediction methods tested on the MIMIC III dataset specifically for SBP and DBP. Notably, Baker et al.^11^ used PPG and ECG waveforms as input signals, similar to our approach, and achieved MAE scores of 4.41 ± 6.11 mmHg for SBP and 2.91 ± 4.23 mmHg for DBP. These are among the lowest scores compared to ours and other deep-learning methods that focused on a single waveform, such as ECG in the work of Miao et al.^20^ or PPG in the studies by Slapnicar et al.^21^, and Schrumpf et al.^22^ However, their SBP and DBP predictions are limited to only 90–130 mmHg and 60–90 mmHg, respectively. Similarly, the lowest DBP prediction performance in T et al.^[23]^have only been validated with 55 subjects of the MIMIC III dataset. Our proposed method demonstrates the highest $r$ metric for both SBP and DBP predictions, at 0.88 and 0.92, respectively. Furthermore, it achieved high performance with low MAE scores at 4.15 ± 5.83 mmHg for SBP predictions and 2.33 ± 3.16 mmHg for DBP predictions. Additionally, our proposed algorithm has the smallest prediction model by size comparison, with only 37,265 in the number of parameters.

**Table 4:** Comparative analysis of the proposed method against previous studies using the MIMIC III dataset.

Work of (Input)	Model parameters	Total subjects	Target measurement (min–max)	Performance metrics						Standards of evaluati		
				${\varvec{r}}$	MAE	SD	CP Δx ≤ 5	CP Δx ≤ 10	CP Δx ≤ 15	BHS grade	AAMI grade	IEEE grade
^21^ (PPG)	Unspecified	510	SBP (**70–200**)	–	9.43	–	–	–	–	–	–	–
			DBP (**30–115**)	–	6.88	–	–	–	–	–	–	–
^20^ (ECG)	404,520	428	SBP **(**80–180**)**	**0.88**	7.1	9.99	50.07	76.40	90.39	B	Pass	D
			DBP (60–100)	0.71	4.61	6.29	65.66	89.77	96.63	A	Pass	A
^11^ (PPG & ECG)	Unspecified	**6,972**	SBP (90–130)	0.80	**4.41**	**6.11**	**67.66**	**89.82**	**96.82**	A	Pass	–
			DBP (60–90)	0.85	2.91	4.23	**82.79**	96.12	99.09	A	Pass	–
^22^ (PPG)	Unspecified	750	SBP (75–165)	–	7.7	–	–	–	–	–	–	–
			DBP (40–80)	–	4.4	–	–	–	–	–	–	–
^22^ (PPG)	Unspecified	55	SBP (80–180)	–	5.34	7.04	63.4	85.9	92.78	B	Pass	B
			DBP (40–80)	–	**2.89**	**3.79**	81.70	**98.28**	**100**	A	Pass	A
Ours (PPG & ECG)	**37,625**	431	SBP (90–180)	**0.88**	5.31	7.25	59.82	85.92	94.93	B	Pass	B
			DBP (50–110)	**0.92**	3.30	4.76	80.04	95.42	98.61	A	Pass	A

This table employs the same evaluation metrics and standards as outlined in Tables 2 and 3, with the addition of model size (defined by parameter count) and total subject count as comparative metrics.

Significant values are in [bold]

## Discussion

In this study, we proposed an algorithm that utilizes hybrid latent features from two pulses of PPG and ECG signals, as well as the R-to-R duration intervals. By using only short-length input signals, we can achieve a model that is small, fast, and computationally efficient. Compared to the previous work of Jeong and Lim^12^, where they merged the PPG and ECG signals by creating another signal, which is the difference between the two signals, we merged the features (not the raw signals) by concatenation and using Conv1D layers and LSTM cells. We observed that features derived from R-to-R duration intervals are particularly effective in enhancing SBP and DBP prediction using the Conv1D model. Furthermore, we examined the effectiveness of LSTM cells in processing these hybrid latent features. The results of our experiments suggest that combining the latent features of PPG and ECG and processing them with LSTM cells, leads to improved performance in BP prediction.

Comparing the performance of previous studies in predicting BP is generally challenging for researchers due to the use of different datasets or subdatasets. For instance, Annunziata et al.^9^ conducted experiments on two distinct datasets: a subset of the MIMIC I dataset and a custom dataset. Their findings suggested that experiments with fewer subjects tend to yield better performance. The MIMIC III dataset, being substantially larger and encompassing a wider variety of patient pathophysiologies, presents a more challenging environment for such predictions. Jeong and Lim^12^ and T et al.^23^ only used 48 subjects of MIMIC I and 55 subjects of MIMIC III, respectively. While having higher metric performances, the robustness of their method remains uncertain when compared to other methods that validate their performance on a dataset with a higher number of subjects. Additionally, the range limit of the blood pressure values, i.e., maximum and minimum SBP and maximum and minimum DBP, should be standardized for comparison. Baker et al.^11^ results may perform well significantly, maybe due to the small range of these values.

Our proposed method has demonstrated a robust performance of SBP and DBP predictions with low error metrics when compared with other studies using the MIMIC III dataset. However, our SBP prediction performance had only nearly achieved the best category, i.e., an A grade, according to the BHS guideline. Additionally, according to the AAMI standards, BP devices should be evaluated using a broader range of values. This includes an SBP range with at least 10% of readings above 180 mmHg, whereas in our experimental data, we had only found a maximum BP value of 180 mmHg. Further experimentation and tweaking of the hyperparameters of this model may be necessary to surpass these limitations.

## Conclusion

In this study, we developed a continuous non-invasive BP prediction system by integrating raw PPG, ECG, and R-to-R duration interval data. The experimental results indicate that the Conv1D-LSTM model achieves the lowest error metrics for SBP and DBP prediction, both with and without the inclusion of R-to-R duration intervals in the hybrid latent feature vector. Compared to previous studies, our proposed system demonstrates superior performance with highest $r$ metrics scores of 0.877 for SBP and 0.918 for DBP predictions, and low MAE scores of 4.15 ± 5.83 mmHg and 2.33 ± 3.16 mmHg for for SBP and DBP predictions, respectively. Tested on 431 patients from the MIMIC III dataset, our method successfully met the evaluation standards of BHS, AAMI, and IEEE. This experiment reveals that the use of hybrid latent features from PPG and ECG, along with R-to-R duration interval data and LSTM cells, enhances the accuracy of SBP and DBP predictions.

## Data Availability

The dataset used in this study can be accessed via the Physionet website at https://physionet.org/content/mimiciii

## References

[1] Hayes S (2019). Comparison of blood pressure measurements in the upper and lower extremities versus arterial blood pressure readings in children under general anesthesia. Med. Dev..

[2] Hager, H. H. & Burns, B. Artery cannulation. StatPearls [Internet]. Treasure Island (FL): StatPearls publishing; -Available from: https://www.ncbi.nlm.nih.gov/books/NBK482242/29489243

[3] Bote JM, Recas J, Hermida R (2020). Evaluation of blood pressure estimation models based on pulse arrival time. Comput. Electr. Eng..

[4] Mukkamala R (2015). Toward ubiquitous blood pressure monitoring via pulse transit time: Theory and practice. IEEE Trans. Biomed. Eng..

[5] Barvik D, Cerny M, Penhaker M, Noury N (2022). Noninvasive continuous blood pressure estimation from pulse transit time: A review of the calibration models. IEEE Rev. Biomed. Eng..

[6] Gesche H, Grosskurth D, Küchler G, Patzak A (2012). Continuous blood pressure measurement by using the pulse transit time: Comparison to a cuff-based method. Eur. J. Appl. Physiol..

[7] Xie Q, Wang G, Peng Z, Lian Y (2018). Machine learning methods for real-time blood pressure measurement based on photoplethysmography. IEEE Int. Conf. Digit. Signal Process..

[8] Tazarv A, Levorato M (2021). A deep learning approach to predict blood pressure from PPG signals. IEEE EMBS.

[9] Paviglianiti A, Randazzo V, Villata S, Cirrincione G, Pasero E (2022). A comparison of deep learning techniques for arterial blood pressure prediction. Cogn. Comput..

[10] Mahmud S (2022). A shallow U-net architecture for reliably predicting blood pressure (BP) from photoplethysmogram (PPG) and electrocardiogram (ECG) signals. Sensors.

[11] Baker S, Xiang W, Atkinson I (2021). A hybrid neural network for continuous and non-invasive estimation of blood pressure from raw electrocardiogram and photoplethysmogram waveforms. Comput. Methods Programs Biomed..

[12] Jeong DU, Lim KM (2021). Combined deep CNN-LSTM network-based multitasking learning architecture for noninvasive continuous blood pressure estimation using differences in ECG-PPG features. Sci. Rep..

[13] Johnson AEW (2016). MIMIC-III, a freely accessible critical care database. Sci. Data..

[14] Pan J, Tompkins WJ (1985). A real-time QRS detection algorithm. IEEE Trans. Biomed. Eng..

[15] Clevert, D. et al. Fast and accurate deep network learning by exponential linear units (ELUs). Preprint at https://arxiv.org/abs/1511.07289 (2015).

[16] Hochreiter S, Schmidhuber J (1997). Long short-term memory. Neural Comput..

[17] O’Brien E (1993). The British hypertension society protocol for the evaluation of blood pressure measuring devices. J. Hypertens..

[18] White WB (1993). National standard for measurement of resting and ambulatory blood pressures with automated sphygmomanometers. Hypertens..

[19] IEEE Standard Association (2019). IEEE standard for wearable, cuffless blood pressure measuring devices – Amendment 1 (Amendment to IEEE Std 1708–2014). IEEE Std. 1708a-2019.

[20] Miao F (2020). Continuous blood pressure measurement from one-channel electrocardiogram signal using deep-learning techniques. Artif. Intell. Med..

[21] Slapničar G, Mlakar N, Luštrek M (2019). Blood pressure estimation from photoplethymogram using a spectro-temporal deep neural network. Sensors..

[22] Schrumpf F, Frenzel P, Aust C, Osterhoff G, Fuchs M (2021). Assessment of non-invasive blood pressure prediction from PPG and rPPG signals using deep learning. Sensors..

[23] Mahardika TNQ, Fuadah YN, Jeong DU, Lim KM (2023). PPG signals-based blood-pressure estimation using grid search in hyperparameter optimization of CNN-LSTM. Diagnostics..

